# Longevity and pleural mesothelioma: age-period-cohort analysis of incidence data from the Surveillance, Epidemiology, and End Results (SEER) Program, 1973–2013

**DOI:** 10.1186/s13104-018-3436-0

**Published:** 2018-05-23

**Authors:** Brent D. Kerger

**Affiliations:** Exponent Inc., 15615 Alton Parkway, Suite 350, Irvine, CA 92618 USA

**Keywords:** Epidemiology, Cohort effect, Period effect, Age effect, Age distribution, Asbestos, World War II era

## Abstract

**Objective:**

This study investigates the hypothesis that an increasing fraction of incident pleural mesothelioma (PM) in the US population may be related to longevity, i.e., to expansion of the population over age 75 years with an age-related elevation in risk. An age-period-cohort analysis of the SEER 9 cancer registries (1973–2013) was conducted using 5-year intervals of age, calendar period, and birth cohort after stratification into four gender-age groups (male and female; 0–74 and 75+ years).

**Results:**

Gender-specific time trends in age-adjusted PM incidence by age groups were observed. After adjusting for cohort effects, males in the 0–74-year age group experienced rapidly declining PM incidence rates following the observed peak in 1978–1982, whereas continuously increasing incidence rates were observed among older males. A significant cohort effect was also observed among males in both age groups, with peak incidence rates in the 1926–1930/1928–1932 birth cohorts and thereafter. The distinct period and cohort effects among males age 0–74 years may be driven by declining age-adjusted PM incidence rates corresponding to the decline in occupational asbestos exposures post-World War II, whereas the increasing time trend seen in both genders at age 75+ may reflect an increasing proportion due to longevity-related factors.

**Electronic supplementary material:**

The online version of this article (10.1186/s13104-018-3436-0) contains supplementary material, which is available to authorized users.

## Introduction

**Fig. 1:**
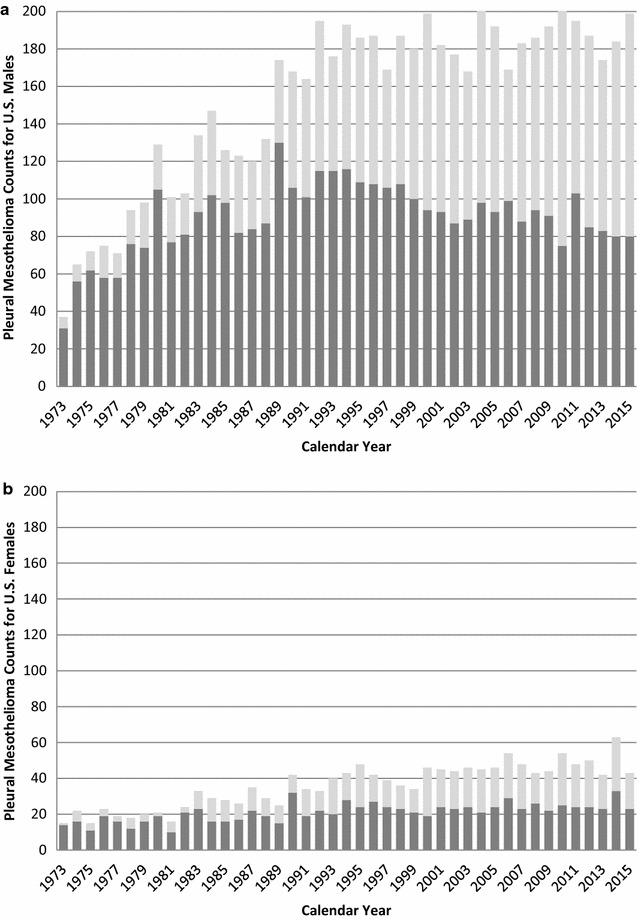
Pleural mesothelioma counts stratified by age (0–74, 75+ years old) among US males (**a**) and females (**b**). Darker portion of each bar corresponds to ages 0–74 years and lighter portion to 75+ years. Based on annual incidence data from the SEER 9 registry collected from 1973 to 2015

The commonly considered etiology of malignant pleural mesothelioma (PM) is occupational asbestos exposure; however, the disease can also arise due to exposures to erionite, non-commercial amphiboles, or ionizing radiation, and from genetic predisposition or spontaneous occurrence [1]. In the past three decades, PM incidence data from the SEER program revealed a peak male age-adjusted rate occurring in the early 1990s and a subsequent decline [2–4]; the decline was more pronounced among males age 0–74 [3, 5, 6]. The SEER 9 data also reveal an increasing proportion of PM among age 75+ males and females since the early 1990s (Fig. 1). The age-adjusted PM decline among age 0–74 males may relate to increasingly stringent US regulations on asbestos use that were put into place starting in the 1970s.

Logically, a fraction of PM incidence at age 75+ must be attributable to decades-prior occupational asbestos exposures among longevity-prone individuals. However, recent reports have identified increased age-adjusted PM incidence for age 75+ males and females [5, 6], raising questions about what fraction occurred spontaneously or from non-asbestos causes. Using linear spline analysis for the SEER 9 data through 2012, Beckerman et al. [6] reported that PM incidence among males age 0–74 is predicted to intersect the rate for age 0–74 females within the next decade.

**Fig. 2:**
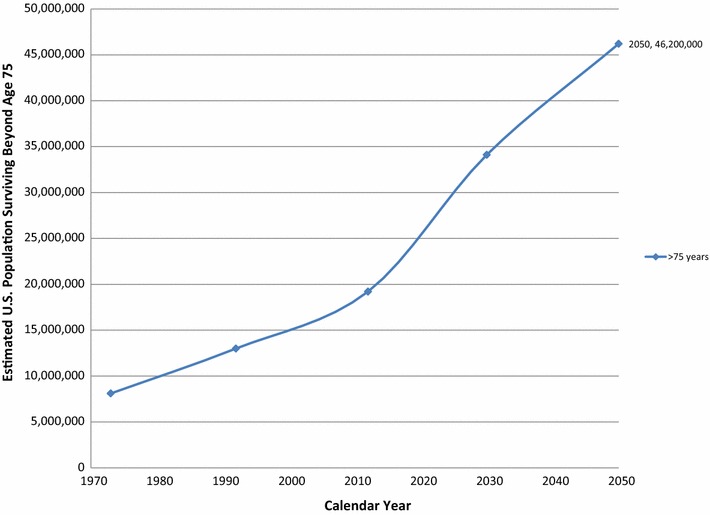
United States residents surviving beyond age 75 for 1973 to 2050. Based on US Census Bureau data and projections for the selected years [7, 8]

Dramatic changes in the actual and predicted age distribution of the US population have been documented in the past three decades, concurrent with the rise in age-adjusted PM incidence for age 75+ males and females (Fig. 2). Based on census data [7, 8], the number of individuals in the US population living beyond age 75 has more than doubled between 1973 and 2012. Because of elevated birth rates in the US between 1946 and 1964 (the so-called ‘baby boomer generation’) and increased longevity, the number of individuals surviving beyond age 75 is predicted to more than double again between 2012 and 2050 [7–9]. Accordingly, longevity-related changes are expected to become increasingly important influences on the incidence of PM and other late-life cancers [5, 6].

It is hypothesized that some of the continued elevation in PM in more elderly individuals (i.e., the 75+ age group) may be due to age-related factors in this growing population subset. This hypothesis is investigated by conducting an age-period-cohort analysis of males and females age 0–74 and age 75+ within the SEER 9 cancer registries (1973–2013).

## Main text

### Methods

The incidence data for primary malignant pleural mesothelioma (International Classification of Diseases for Oncology, 3rd edition, histology codes 9050-9055, sites C38.4 and C38.8) was obtained from the US Surveillance, Epidemiology, and End Results (SEER) 9 population-based cancer registries from 1973 to 2013. Data were extracted by 5-year age and calendar-year groups; due to the need for evenly grouped categories (e.g., 1973–1977, 1978–1982 … 2008–2012), the year 2013 was omitted from period analyses. All analyses were stratified by selected age category (0–74 or 75+ years) and sex (male or female). Data were accessed using SEER*Stat software version 8.3.2 after execution of the SEER data use agreement which includes compliance with ethical and privacy considerations and allows use of the cancer incidence data without separate requirements for study subject consent or Institutional Review Board approval.

The National Cancer Institute (NCI) web tool for age-period-cohort (APC) analysis was applied as described by Rosenberg et al. [10]. The NCI APC web tool enables analysis of net drift (annual percentage change in the expected age-adjusted rates over time), local drifts (annual percentage change in the expected age-specific rates over time), fitted temporal trends (expected rates over time in the reference age group, adjusted for cohort effects), cross-sectional age curve (expected age-specific rates in the reference calendar period, adjusted for cohort effects), longitudinal age curve (expected age-specific rates in the reference birth cohort, adjusted for period effects), period rate ratios (ratio of age-specific rates in each calendar period relative to the reference period), and cohort rate ratios (ratio of age-specific rates in each birth cohort relative to the reference cohort). The NCI APC web tool also enables statistical testing of several null hypotheses related to the stability, log-linearity, and equality of observed trends. Default reference groups were used for comparisons, i.e., the median calendar period (1988–1992) and the median birth cohorts (1931–1935 for ages 0–74; 1906–1910 for ages 75+).

The age categories of 0–74 and 75+ years were selected based on demographics pertaining to post-retirement longevity and age-related PM trends associated with occupational asbestos exposure. First, age 75+ can be considered an ‘old age’ category, given that it is approximately a decade beyond the common retirement age (between 62 and 67 years) for US workers during the 1990s and 2000s. Second, the peak age-specific incidence and mortality from PM in the US is estimated to be between ages 70 and 80 [11]; age 75 represents a median point higher than the mean age at PM death in the US (72.8 ± 11.1 years) and worldwide (70.1 ± 11 years) based on data from 1994 to 2008 [12]. Third, among German asbestos workers the mean age at mesothelioma diagnosis was 60.4 ± 9.9 years and mean age at mesothelioma death was 63.6 ± 10.7 years in 1987–1999 [13]. This indicates that the vast majority of cases due to occupational asbestos exposure occurred in the 0–74 age category. Similar findings for PM death within the 0–74 age range were reported for US insulation workers [14].

### Results

Key findings of the APC analysis for PM in the four age/gender groups are summarized in Table 1. The period effect after adjustment for birth cohort effects revealed similar peak PM incidence years according to age group: younger females showed a peak value in 1973–1977 and younger males peaked slightly later in 1978–1982; older males and females both showed peak incidence in the most recent 5-year time period (2008–2012) (Table 1; Additional file 1: Figure S1). Net drift values indicated significant decreasing time trends in age-adjusted PM incidence for age 0–74 males but not for age 0–74 females, while increasing net drift trends were found for males and females age 75 + (Table 1). Period rate ratios (in comparison with the reference period of 1988–1992) were significantly different from 1.0 for all groups except age 0–74 females (Table 1). Analysis of period deviations indicated significant non-linear trends for males of both age groups, but no significant deviation from log-linearity for females (Table 1).

**Table 1:** Summary of age-period-cohort analysis for pleural mesothelioma incidence in four selected gender/age groups

APC parameters	Males, age 0–74	Males, age 75+	Females, age 0–74	Females, age 75+
Period effect: peak PM				
Incidence years	1978–1982	2008–2012	1973–1977	2008–2012
Net drift (95% CI)	− 1.8% (− 2.3%, − 1 3%)	+4.0% (3.2%, 4.7%)	− 1.5% (− 3.3%, 0.3%)	+3.7% (1.7%, 5.7%)
p value	< 0.0001	< 0.0001	0.11	0.0002
Period RRs different from 1988 to 1992?	Yes	Yes	No	Yes
p value	< 0.0001	< 0.0001	0.67	0.02
Period deviation is non-linear?	Yes	Yes	No	No
p value	0.03	0.05	0.89	0.81
Cohort effect: peak PM				
Birth cohort years	1928–1932	1928–1932	1931–1935	1921–1925
Local drifts = net drift for all age groups?	No	No	No	Yes
p value	< 0.0001	0.0002	0.02	0.58
Cohort RRs different from referent cohort?	Yes	Yes	No	Yes
p value	< 0.0001	< 0.0001	0.16	0.01
Cohort deviation is nonlinear?	Yes	Yes	No	No
p value	<0.0001	<0.0001	0.16	0.91
Cross-sectional age trend (95% CI)	12.5% (11.8, 13.2%)	2.2% (0.9, 3.5%)	7.2% (5.9, 8.4%)	− 0.1% (− 4.1, 3.9%)
Longitudinal age trend (95% CI)	10.7% (9.9, 11.4%)	− 1.7% (− 3.1, − 0.4%)	8.7% (6.7, 10.6%)	− 3.7% (− 7.8, 0.4%)
Longitudinal vs. cross-sectional RR trend	Negative	Positive	Negative	Positive/flat
Age deviation is non-linear?	No	Yes	No	No
p value	0.41	0.0002	0.46	0.07

The birth cohort effect after adjustment for period effects revealed that peak PM incidence occurred in comparable birth cohort years for both genders according to age group. Age 0–74 females showed peak PM incidence in the 1931–1935 birth cohort, just after the peak (1928–1932 birth cohort) for age 0–74 males. Age 75+ females showed a peak PM incidence in the 1921–1925 birth cohort, just prior to the peak (1926–1930) for age 75+ males (Table 1; Additional file 2: Figure S2). Net drift (age-adjusted time trend) was not significantly different from local drift (age-specific time trend) in any group except age 75+ females (Table 1). Cohort rate ratios were significantly different from 1.0 in comparison with the reference group (1931–1935 for ages 0–74; 1906–1910 for ages 75+), and cohort deviations indicated significant non-linearity, except among age 0–74 females, for whom no significant cohort effect was detected (Table 1).

Analysis of age effects on PM incidence after adjustment for period or birth cohort effects was accomplished by examining cross-sectional and longitudinal age trends. Both age effects were stronger for age 0–74 males (cross-sectional age trend 12.5%; longitudinal age trend 10.7%) than for females (7.2 and 8.7%, respectively), whereas a slight positive or no significant age effect was observed among age 75+ males (2.2% and − 1.7%, respectively) and females (− 0.1 and − 3.7%, respectively). Distinct negative trends consistent with a negative net drift were observed in the longitudinal versus cross-sectional rate ratio analysis for age 0–74 groups in both genders, whereas positive trends consistent with a positive net drift were observed for both genders at age 75+ (Additional file 3: Figure S3). Analysis of age deviations revealed significant non-linearity in age 75+ males and marginally significant (p = 0.07) non-linearity for age 75+ females, but no significant non-linearity in the age 0–74 groups (Table 1).

### Discussion

This APC analysis of US SEER 9 cancer registry data from 1973 to 2013 demonstrates significant age, period, and birth cohort effects consistent with longevity-related factors since the early 1990s playing a progressively more important role in PM incidence among US males and females (Fig. 1). Analysis of four age-gender groups revealed distinct trends in PM incidence between males and females age 0–74 or age 75+ that are masked beneath the total age-adjusted PM incidence among US males, which has declined considerably since the early 1990s [2–6].

Most notable are gender-specific differences in age, period, and cohort trends potentially associated with the much higher frequency of occupational/military asbestos exposures expected for males. Specifically, the results revealed that PM incidence in the 0–74 age group has declined since the early 1990s for males, whereas it has increased for both genders in the 75+ age group (Additional file 3: Figure S3). The birth cohorts corresponding to peak PM incidence were nearly identical for males age 0–74 (1928–1932 birth cohort) and age 75+ (1926–1930 birth cohort), while the peak for females age 0–74 (1931–1935 birth cohort) was 10 years later than that for age 75 + (1921–1925 birth cohort), as shown in Additional file 2: Figure S2. Moreover, the analysis of period effects showed that peak PM incidence for age 0–74 males (1978–1982) occurred just after that for females of the same age (1973–1977), whereas the age 75+ males and females both showed gradually increasing PM incidence with a peak in the most recent 5-year period (Additional file 1: Figure S1). Thus, the 1992 peak in total age-adjusted PM incidence among US males previously observed in other analyses resulted from the superposition of the gradual increase in older males (age 75+) and the declining trend since 1978–1982 among younger males (age 0–74). The common birth cohort for peak PM incidence in males of both age groups (but not in females) is consistent with a prominent influence of occupational/military asbestos exposures during the World War II era (i.e., 1940–1950) on male PM incidence trends. The fraction of female PM incidence attributable to World War II era occupational or para-occupational asbestos exposures is unknown, but the low magnitude and relatively flat total incidence trends over the past four decades suggest a limited impact. Further, the earlier period effect peak among age 75+ females and the parallel increasing trends in age-adjusted PM incidence for both genders suggest that other factors relating to longevity may better explain these trends for older individuals.

Overall, our findings are consistent with those of European studies where the temporal and birth cohort trends have been linked to periods of peak occupational asbestos exposure and consumption surrounding World War II and subsequent rebuilding (see Additional file 4: Additional discussion). These studies collectively suggest a plausible impact of longevity-related factors on PM incidence which should be considered when projecting future PM rates attributable to occupational asbestos exposures and other known causes and risk factors.

## Limitations

The primary limitation of this study is the lack of direct linkage data for assessing individual risk of PM related known causes (e.g., exposures to asbestos, erionite, or radiation) versus more general longevity-related factors (e.g., aging, spontaneous disease occurrence, and misclassification or enhanced detection). Some of the age-gender-time categories analyzed may have been too small to provide statistically stable APC results, particularly in regards to the lower PM incidence among females.

## Additional files

**Additional file 1: Figure S1.** Graphic presentation of APC data illustrating the period effect after adjustment for birth cohort effects on PM incidence in SEER 9 registries (1973–2013) in males age 0–74 (Panel A), males age 75+ (Panel B), females age 0–74 (Panel C) and females age 75+ (Panel D). Rate ratios significantly different from 1.0 were identified for A (p < 0.0001), B (p < 0.0001), and D (p = 0.02), but not for C (p = 0.67). Period deviations indicate significant non-linearity for A and B, but not for C and D.

**Additional file 2: Figure S2.** Graphic presentation of APC data illustrating the cohort effect after adjustment for period effects on PM incidence in SEER 9 registries (1973–2013) in males age 0–74 (Panel A), males age 75+ (Panel B), females age 0–74 (Panel C) and females age 75+ (Panel D). Rate ratios significantly different from 1.0 were identified for A (p < 0.0001), B (p < 0.0001), and D (p = 0.01), but not for C (p = 0.16). Cohort deviations indicate significant non-linearity for A, B, and D, but not for C.

**Additional file 3: Figure S3.** Graphic presentation of APC data illustrating the longitudinal versus cross-sectional age effect on PM incidence in SEER 9 registries (1973–2013) in males age 0–74 (Panel A), males age 75+ (Panel B), females age 0–74 (Panel C) and females age 75+ (Panel D). Changes in net drift are consistent with the opposing slopes for the age 0–74 (negative) versus age 75+ (positive) rate ratios.

**Additional file 4: Additional discussion.** Further detailed interpretation of relevant scientific literature that was beyond the length limitations for the main manuscript.

## Abbreviations

APC: age-period-cohort
NCI: National Cancer Institute
PM: pleural mesothelioma
SEER: Surveillance, Epidemiology, and End Results Program
US: United States
