# Supplementary material for: Longevity and pleural mesothelioma: age-period-cohort analysis of incidence data from the Surveillance, Epidemiology, and End Results (SEER) Program, 1973–2013
Source: BMC Res Notes. 2018 May 23;11:337. doi: 10.1186/s13104-018-3436-0 (PMC5966894; doi:10.1186/s13104-018-3436-0)
Supplement: Supplementary file 2 — Additional file 2: Figure S2. Graphic presentation of APC data illustrating the cohort effect after adjustment for period effects on PM incidence in SEER 9 registries (1973–2013) in males age 0–74 (Panel A), males age 75+ (Panel B), females age 0–74 (Panel C) and females age 75+ (Panel D). Rate ratios significantly different from 1.0 were identified for A (p < 0.0001), B (p < 0.0001), and D (p = 0.01), but not for C (p = 0.16). Cohort deviations indicate significant non-linearity for A, B, and D, but not for C. [file 13104_2018_3436_MOESM2_ESM.pdf]

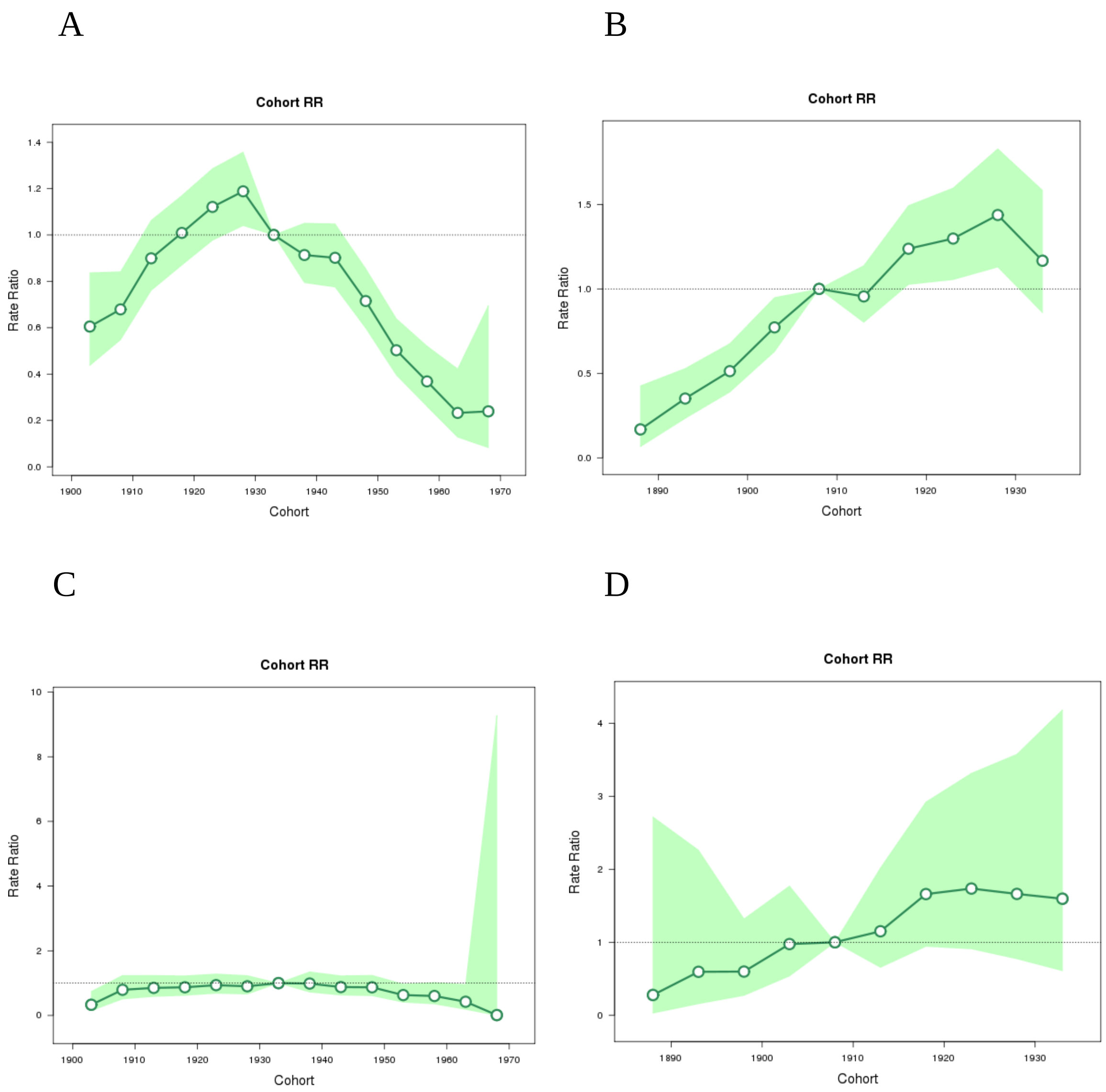

Supplemental Figure S-2. Cohort effect after adjustment for period effects on PM incidence in SEER 9 registries (1973-2013) in males age 0-74 (Panel A), males age 75+ (Panel B), females age 0-74 (Panel C) and females age 75+ (Panel D). Rate ratios significantly different from 1.0 were identified for A ($p < 0.0001$), B ($p < 0.0001$), and D ($p = 0.01$), but not for C ($p = 0.16$). Cohort deviations indicate significant non-linearity for A, B, and D, but not for C.
