# Supplementary material for: Longevity and pleural mesothelioma: age-period-cohort analysis of incidence data from the Surveillance, Epidemiology, and End Results (SEER) Program, 1973–2013
Source: BMC Res Notes. 2018 May 23;11:337. doi: 10.1186/s13104-018-3436-0 (PMC5966894; doi:10.1186/s13104-018-3436-0)
Supplement: Supplementary file 3 — Additional file 3: Figure S3. Graphic presentation of APC data illustrating the longitudinal versus cross-sectional age effect on PM incidence in SEER 9 registries (1973–2013) in males age 0–74 (Panel A), males age 75+ (Panel B), females age 0–74 (Panel C) and females age 75+ (Panel D). Changes in net drift are consistent with the opposing slopes for the age 0–74 (negative) versus age 75+ (positive) rate ratios. [file 13104_2018_3436_MOESM3_ESM.pdf]

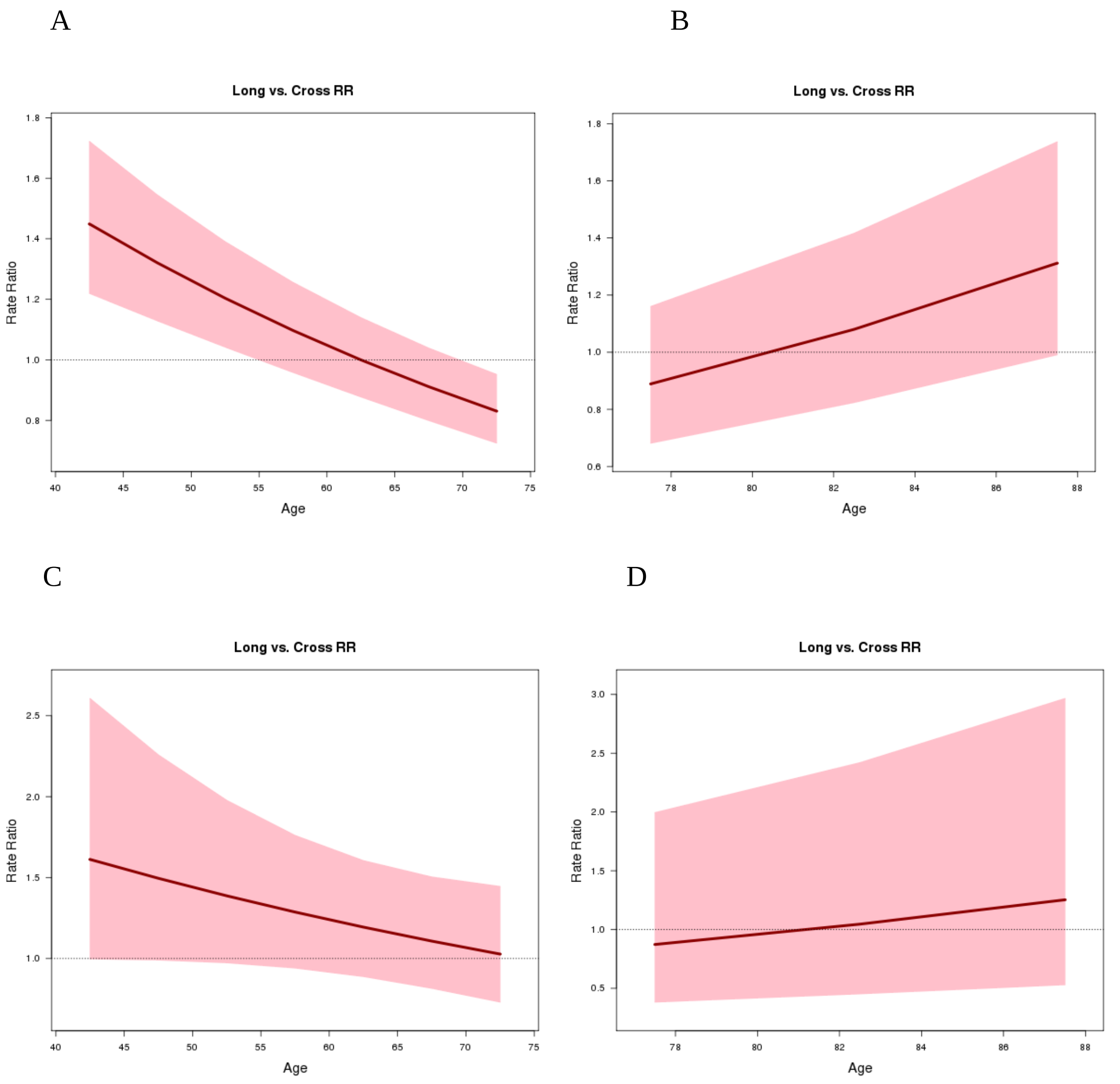


**Supplemental Figure S-3. Longitudinal versus cross-sectional age effect on PM incidence in SEER 9 registries (1973-2013) in males age 0-74 (Panel A), males age 75+ (Panel B), females age 0-74 (Panel C) and females age 75+ (Panel D).** Changes in net drift are consistent with the opposing slopes for the age 0-74 (negative) versus age 75+ (positive) rate ratios.
