# Supplementary material for: Longevity and pleural mesothelioma: age-period-cohort analysis of incidence data from the Surveillance, Epidemiology, and End Results (SEER) Program, 1973–2013
Source: BMC Res Notes. 2018 May 23;11:337. doi: 10.1186/s13104-018-3436-0 (PMC5966894; doi:10.1186/s13104-018-3436-0)
Supplement: Supplementary file 4 — Additional file 4: Additional discussion. Further detailed interpretation of relevant scientific literature that was beyond the length limitations for the main manuscript. [file 13104_2018_3436_MOESM4_ESM.docx]

**Additional file 4: Additional discussion**

Projections of occupational asbestos-related PM (and background/non-asbestos cases) have been driven by assumptions of relatively high asbestos exposures to the large US workforce involved in shipbuilding and shipyard repairs in the World War II era (Lilienfeld et al., 1988; Walker et al., 1983; Nicholson et al., 1982). A focus on this era is also supported by the more prominent use of amosite for ship insulation (Franke and Paustenbach, 2011; Teta et al., 2008; Rushworth, 2005) because this amphibole form is a highly potent cause of mesothelioma (Hodgson and Darnton, 2001; Berman and Crump, 2008a, 2008b). The timing of a birth cohort’s first entry into the workforce at age 18 can be simulated by adding 18 years to the birth cohort range. For example, adding 18 years to the male birth cohort range for peak PM incidence (1926-1932 for both age groups) leads to a calendar period (1944-1950) plausibly circumscribing the period of more intense post-World War II shipyard production and ship repair activities. Increasing recognition and control of asbestos hazards and associated declining amosite exposures (Franke and Paustenbach, 2011; Rushworth, 2005) plausibly corresponds with reduced PM incidence among persons with first occupational exposure after the 1950s in US shipyards.

Nuances in the period and birth cohort trends in PM incidence may also provide useful insights, particularly among males as a result of their more robust data. First, a relatively monotonic decline in PM incidence rate by calendar period (adjusted for birth cohort effects) is observed in age 0-74 males following the peak in 1978-1982 calendar years (Supplemental Figure S-1) and a relatively monotonic decline is observed in this group following the birth cohort peak in 1928-1932 (Supplemental Figure S-2). Adding 18 years to this birth cohort corresponds to an early workforce exposure period of 1946-1950 and an associated latency to peak PM rate ratio (in 1978-1982) of perhaps 28 to 36 years. This latency range is consistent with other estimates for PM caused by high occupational exposures to amphibole asbestos among mining and milling workers, shipyard workers, insulators, and pipefitters (Neumann et al., 2001; Bianchi et al., 1997; Lanphear and Buncher, 1992; Selikoff et al., 1980).

Also of note in the birth cohort analysis is an apparent flattening of the PM incidence rate for age 0-74 males after a monotonic decline starting in the early 1960s (Supplemental Figure S-2). Adding 18 years to the birth cohort preceding the flattened trend (1954-1958) corresponds to an early workforce exposure period of 1972-1976, when more stringent regulations on workplace asbestos exposure were invoked in the US. Also shown in Supplemental Figure S-2 is an apparent flattening or decline in the PM incidence rate among age 75+ males after the 1928-1932 birth cohort. Adding 18 years to the latter birth cohort corresponds to an early workforce exposure period of 1946-1950 within the peak of post-World War II shipbuilding and repairs. However, the peak PM incidence rate for age 75+ males in 1926-1930 is small and may represent a plateau, as discussed below. If this peak was related to occupational asbestos exposures it would correspond to an unusually long average latency of 60-68 years, which appears to be rare for occupational asbestos exposures (Reid et al., 2014; Frost, 2013; Lacourt et al., 2012; Marinaccio et al., 2012; Le Stang et al., 2010; Marinaccio et al., 2007; Neumann et al., 2001; Bianchi et al., 1997; Lanphear and Buncher, 1992; Selikoff et al., 1980). Notably, Lacourt et al. (2012) reported that occupational asbestos exposures under age 20 were a critical risk factor for PM in France, and the PM risk plateaued at 30 years after cessation of exposure.

The distinctly different PM trends for age 0-74 males when compared to age 75+ in both genders are consistent with a shift towards later age at PM onset for both genders in the US (Figure 1). This shift corresponds with the increasing size and proportion of the US population over age 75 (Figure 2) and is likely to be of increasing importance in the next few decades as the ‘baby boomer’ generation continues to fill the ranks of the older US population. The SEER 9 data for age 75+ groups also suggest that PM risk drops off substantially among those surviving longest, e.g., for persons surviving beyond age 85. This trend may be associated with greater resistance to cancer among the most longevity-prone individuals.

Findings compatible with the current study were reported in earlier analyses of PM incidence using the US SEER program data (Moolgavkar et al., 2009; Price and Ware, 2009). The current study adds to these earlier analyses with extended SEER 9 data that discern the decline in PM incidence since 1978-1982 among age 0-74 males and the concurrently increasing PM incidence among age 75+ males and females.

European researchers have reported shifting trends in PM incidence or mortality in the last two decades in Italy (Girardi et al., 2014; Mensi et al., 2016), France (Lacourt et al., 2012; LeStang et al., 2010), Belgium (Van den Borre and Deboosere, 2014), Sweden (Jarvholm and Burdorf, 2015), the Netherlands (Segura et al., 2003), and the United Kingdom (Tan et al., 2010). Studies examining age-specific trends are discussed further below. The extent to which these shifting PM trends are due to region-specific variation in occupational asbestos exposures and/or longevity-related factors is currently unknown.

Based on APC analysis, Girardi et al. (2014) found that temporal trends in Italy correlated with historical asbestos consumption and PM incidence was explained mostly by an age-cohort effect with highest risk among oldest age-classes (peak at age 74-79) and for cohorts born around 1940. Girardi et al. (2014) observed a significant decline in PM incidence among younger age classes (i.e., 50-54 and 55-59) starting in 1999-2002, supporting their prediction that PM incidence among those under age 75 will be more than halved in the next 16 years as reported for other European countries (Tan et al., 2010; Segura et al., 2003). Girardi et al. (2014) also predicted that due to aging of the population in the next 10-15 years the burden of PM among older age groups will remain high. Similarly, Mensi et al. (2016) reported annual PM increases in Italy among older age groups (+3.4 to +3.5% for those over age 65) and concurrent decreases were observed in younger age groups (-3.0 to -4.3% for those under age 65).

The attrition of PM risk among younger age groups and the shift towards higher age-adjusted risks in older age groups is apparent in other European countries as well. Tan et al. (2010) used Bayesian methods to predict peak (total) mesothelioma mortality occurring in 2016 in the UK, with a rapid decline thereafter. Birth cohorts after 1965 showed consistently low numbers of actual and projected mesothelioma deaths, and a steady linear rate of decline was noted for birth cohorts between 1955 and 1965 (Tan et al., 2010). Jarvholm and Burdorf (2015) reported that age-adjusted PM mortality trends for all age groups in Sweden tended to mask a strong influence of PM among long-surviving individuals from more exposed birth cohorts (e.g., 1935 to 1949) that was offset by the rapidly decreasing PM rates among younger birth cohorts. Segura et al. (2003) reported that an age-cohort model among men in the Netherlands showed the highest age-specific death rates among the oldest age group (age 75 to 84) and the highest relative risks for birth cohorts of 1938 to 1947. Segura et al. (2003) noted that the strong increase in male PM incidence between 1969 and 1998 may be affected by increasing diagnostic awareness of mesothelioma since the late 1970s.

**Conclusions**

The current study identifies distinct period and cohort effects on PM incidence trends among US males that are consistent with a prominent influence of high occupational exposures to amosite during the era of World War II. Temporal trends of age-adjusted total PM incidence, which peaked in the early 1990s, apparently masked the peak PM incidence among age 0-74 males in 1978-1982 that was offset by an increased age-adjusted PM incidence in subsequent years among older males. The substantial decline in PM incidence among age 0-74 males after 1982 suggests a decline in PM risk with post-World War II reductions in occupational amosite exposures, consistent with trends observed in other developed countries. By contrast, both males and females in the age 75+ group showed increasing PM incidence in the last two decades. This pattern may correspond to longevity-related factors which plausibly include enhanced PM surveillance, improved PM diagnostic accuracy, increased observation of spontaneous PM in old age, and possible non-occupational causes for PM (e.g., ionizing radiation and exposures to erionite or non-commercial amphiboles; Kerger et al. 2014a). An undefined fraction of age 75+ PM incidence is attributable to occupational and para-occupational asbestos exposures, but attrition of the at-risk population with exposures prior to more stringent regulations (pre-1970s) is likely to continually reduce this fraction (Price and Ware, 2009). Overall, our findings are consistent with those of European studies where the temporal and birth cohort trends have been linked to periods of peak occupational asbestos exposure and consumption surrounding World War II and subsequent rebuilding. These studies collectively suggest a plausible impact of longevity-related factors on PM incidence which should be considered when projecting future PM rates attributable to occupational asbestos exposures and other known causes and risk factors.

References

Berman, DW, and Crump, KS. 2008a. A Meta-Analysis of Asbestos-Related Cancer Risk that Addresses Fiber Size and Mineral Type. Crit Rev Toxicol 38(S1):49-73.

Berman, DW, and Crump, KS. 2008b. Update of Potency Factors for Asbestos-Related Lung Cancer and Mesothelioma. Crit Rev Toxicol 38(S1):1-47.

Bianchi, C, Giarelli, L, Grandi, G, Brollo, A, Ramani, L, Zuch, C. 1997. Latency periods in asbestos-related mesothelioma of the pleura. Eur J Cancer Prev 6:162-166.

Franke K, Paustenbach D. 2011 .Government and Navy knowledge regarding health hazards of asbestos: a state of the science evaluation (1900 to 1970). Inhal Toxicol, 23 Suppl 3, 1-20.

Frost, G. 2013. The latency period of mesothelioma among a cohort of British asbestos workers (1978-2005). Brit J Cancer 109:1965-1973.

Girardi, P, Bressan, V, Merler, E. 2014. Past trends and future prediction of mesothelioma incidence in an industrialized area of Italy, the Veneto Region. Cancer Epid 38:496-503.

Hodgson, JT, Darnton A. 2000. The quantitative risks of mesothelioma and lung cancer in relation to asbestos exposure. Ann Occup Hyg. 2000 Dec;44(8):565-601.

Jarvholm, B, Burdorf, A. 2015. Emerging evidence that the ban on asbestos use is reducing occurrence of pleural mesothelioma in Sweden. Scan J Public Health 43:875-881.

Lanphear, BP, Buncher, CR. 1992. Latent period for malignant mesothelioma of occupational origin. J Occup Med 34:718-721.

Le Stang, N, Belot, A, Gilg Soit Ilg, A, Rolland, P, Astoul, P, Bara, S, Brochard, P, Danzon, A, Dalafosse, P, Grosclaude, P, Gulzard, A-V, Imbernon, E, Lapotre-Ledoux, B, Ligier, K, Molinie, F, Pairo, J-C, Sauleau, E-A, Tretarre, B, Velten, M, Bossard, N, Goldberg, M, Launoy, G, Galateau-Salle, F. 2010. Evolution of pleural cancers and malignant pleural mesothelioma incidence in France between 1980 and 2005. Int J Cancer 126:232-238.

Lilienfeld, DE, Mandel, JS, Coin, P, Schuman, LM. 1988. Projection of asbestos related disease in the United States, 1985-2009 I. Cancer. Brit J Indust Med 45:283-291.

Marinaccio, A, Binazzi, A, Cauzillo, G, Cavone, D, De Zotti, R, Ferrante, P, Gennaro, V, Gorini, G, Menegozzo, M, Mensi, C, Merler, E, Mirabelli, D, Montanaro, F, Musti, M, Pennelli, F, Romanelli, A, Scarselli, A, Tumino, R, Italian Mesothelioma (ReNaM) Working Group. 2007. Eur J Cancer:2722-2728.

Marinaccio, A, Binazzi, A, Di Marzio, D, Scarselli, A, Verardo, M, Mirabelli, D, Gennaro, V, Mensi, C, Riboldi, L, Merler, E, De Zotti, R, Romanelli, A, Chellini, E, Silvestri, S, Pascucci, C, Romero, E, Menegozzo, S, Musti, M, Cavone, D, Cauzillo, G, Tumino, R, Nicita, C, Melis, M, Iavicoli, S, ReNaM Working Group. 2012. Int J Cancer 130:2146-2154.

Mensi, C, De Matteis, S, Dallari, B, Riboldi, L, Bertazzi, PA, Consonni, D. 2016. Indicence of mesothelioma in Lombardy, Italy: exposure to asbestos, time patterns and future projections. Occup Environ Med 73:607-613.

Reid, A., de Klerk, NH, Magnani, C, Ferrante, D, Berry, G, Musk, AW, Merler, E. 2014. Mesothelioma risk after 40 years since first exposure to asbestos: a pooled analysis. Thorax 69:843-850.

Rushworth, DH. 2005. The Navy and asbestos thermal insulation. Naval Eng J 117:35-48.

Segura, O, Burdorf, A, Looman, C. 2003. Update of predictions of mortality from pleural mesothelioma in the Netherlands. Occup Environ Med 60:50-55.

Selikoff IJ, Hammond EC, Seidman H. 1979. Mortality experience of insulation workers in the United States and Canada, 1943--1976. Annals NY Acad Sci, 330, 91-116.

Selikoff, IJ, Cuyler, E, Hammond, EC, Seidman, H. 1980. Latency of asbestos disease among insulation workers in the United States and Canada. Cancer 46:2736-2740

Tan, E, Warren, N, Darnton, AJ, Hodgson, JT. 2010. Projection of mesothelioma mortality in Britain using Bayesian methods. Brit J Cancer 103:430-436.

Van den Borre, L, Deboosere, P. 2014. Asbestos in Belgium: an underestimated health risk. The evolution of mesothelioma mortality rates (1969-2009). Int J Occup Environ Health 20:134-140.

Walker, AM, Loughlin, JE, Friedlander, ER, Rothman, KJ, Dreyer, NA. 1983. Projections of asbestos-related disease 1980-2009. J Occupa Med 25:409-425.
